# Belatacept Conversion in Simultaneous Pancreas‐Kidney and Liver‐Kidney Transplant Recipients: A Single‐Center Feasibility Cohort and Narrative Review

**DOI:** 10.1111/ctr.70719

**Published:** 2026-10-18

**Authors:** Dusan Harmacek, Elena Rho, Seraina von Moos, Severin Simmler, Thomas Welte, Fabian Rössler, Thomas Schachtner, Lukas Weidmann

**Affiliations:** ^1^ Division of Nephrology University Hospital Zurich Zurich Switzerland; ^2^ Division of Nephrology Cantonal Hospital Lucerne Lucerne Switzerland; ^3^ Department of Surgery and Transplantation University Hospital Zurich Zurich Switzerland

**Keywords:** belatacept conversion, costimulation blockade, liver‐kidney transplantation, simultaneous pancreas‐kidney transplantation

## Abstract

**Background:**

Calcineurin inhibitor (CNI) nephrotoxicity drives long‐term kidney‐function decline after simultaneous pancreas‐kidney (SPK) and liver‐kidney (LK) transplantation, but belatacept conversion, as an alternative strategy, is poorly studied in these recipients, whose non‐kidney allograft rejection risk differs.

**Methods:**

We retrospectively studied six SPK and six LK recipients converted from CNI‐ to belatacept‐based immunosuppression and analyzed the cohorts separately alongside a narrative review of published SPK/LK conversion experiences. Outcomes were estimated glomerular filtration rate (eGFR) trajectories and slopes, donor‐specific antibodies (DSA) and rejection, pancreatic, hepatic and metabolic parameters, infections, and survival, reported descriptively with exploratory estimates and 95% confidence intervals (CI).

**Results:**

Conversion occurred a median of 64 (SPK) and 89 (LK) months after transplantation, always with a transient low‐dose CNI overlap (target trough 3‐4 ng/ml). In SPK recipients, median eGFR rose from 45.4 to 55.5 mL/min/1.73m^2^ and the median eGFR slope changed from −3.9 to +5.6 mL/min/year (mixed‐model slope change +12.6, 95% CI 2.9 to 22.3); in LK recipients, from 38.8 to 46.3 and from −3.1 to +4.1 (estimate +6.3, 95% CI −4.2 to 16.7). No new DSA developed; pre‐existing DSA declined or remained stable. Pancreatic and liver function stayed stable. Two infections in LK recipients (*Pneumocystis* pneumonia, adenovirus) were possibly belatacept‐aggravated. Over a median 25 months, 8/12 (67%) remained on belatacept.

**Conclusions:**

Late belatacept conversion with a transient low‐dose CNI overlap was feasible in selected SPK and LK recipients and was associated with stable‐to‐improved kidney function without a pancreatic or hepatic safety signal. The uncontrolled design supports feasibility, not efficacy.

**Abbreviations**
ADPKD: autosomal‐dominant polycystic kidney disease
AMR: antibody‐mediated rejection
ATG: anti‐thymocyte globulin
AZA: azathioprine
Bela: belatacept
BK: BK polyomavirus
caAMR: chronic‐active antibody‐mediated rejection
CI: confidence interval
CKD‐EPI: Chronic Kidney Disease Epidemiology Collaboration
CMV: cytomegalovirus
CNI: calcineurin inhibitor
COVID‐19: coronavirus disease 2019
CTOT: Clinical Trials in Organ Transplantation
CyA: cyclosporine
DBD: donation after brain death
DGF: delayed graft function
DM: diabetes mellitus
dnDSA: de novo donor‐specific antibody
DSA: donor‐specific antibody
EBV: Epstein‐Barr virus
eGFR: estimated glomerular filtration rate
ESRD: end‐stage renal disease
EVR: everolimus
FU: follow‐up
HbA1c: glycated hemoglobin
HCV: hepatitis C virus
HLA: human leukocyte antigen
IQR: interquartile range
IVIG: intravenous immunoglobulin
KAL: kidney‐after‐liver transplantation
LK: liver‐kidney transplantation
LT: liver transplantation
MFI: mean fluorescence intensity
MMDx: Molecular Microscope Diagnostic System
MPA: mycophenolate
mTOR‐I: mammalian target of rapamycin inhibitor
PAK: pancreas‐after‐kidney transplantation
PCP: Pneumocystis jirovecii pneumonia
PCR: polymerase chain reaction
PDN: prednisone
PTA: pancreas transplant alone
PTLD: post‐transplant lymphoproliferative disease
SPK: simultaneous pancreas‐kidney transplantation
Tac: tacrolimus
TMA: thrombotic microangiopathy
TPL: transplantation
UPCR: urine protein‐to‐creatinine ratio

## 1 Introduction

Calcineurin inhibitors (CNIs) anchor maintenance immunosuppression after solid‐organ transplantation, but their nephrotoxicity is a leading cause of progressive kidney dysfunction and graft loss [1]. The problem is hardest to solve in multi‐organ recipients. In simultaneous pancreas‐kidney (SPK) and liver‐kidney (LK) transplantation the kidney carries the same CNI burden as in kidney‐alone recipients, yet the need to protect a second allograft limits CNI minimization [1].

Belatacept, a selective T‐cell costimulation blocker that binds CD80 and CD86 to interrupt CD28 signaling, was developed as a CNI‐free alternative without nephrotoxicity [2] and produced better long‐term kidney function and a 43% lower risk of death or graft loss at 7 years than cyclosporine in *de novo* kidney transplant recipients [3].

The two non‐kidney allografts, however, differ in alloimmune risk. The pancreas is highly immunogenic; rejection is frequent, often clinically silent and carries substantial risk of graft loss [4], and in the randomized Clinical Trials in Organ Transplantation (CTOT)‐15 trial, planned CNI withdrawal under belatacept after *de novo* SPK transplantation was halted for excess pancreas rejection clustered around CNI cessation [5]. The liver, in contrast, protects a simultaneously transplanted kidney from the same donor, with less cellular and antibody‐mediated rejection than in kidney‐alone recipients [6], a protection that does not extend to kidneys transplanted sequentially after a liver [7]. Yet in *de novo* liver transplantation a phase II trial of belatacept‐based primary immunosuppression was stopped early because of excess death, graft loss and post‐transplant lymphoproliferative disease (PTLD), leading to a boxed warning [8]. Both signals arose in *de novo* settings through different mechanisms; whether they apply to late conversion of a stable allograft is unknown, and the two populations warrant separate consideration.

In kidney‐alone transplantation, converting stable recipients from a CNI to belatacept raises the estimated glomerular filtration rate (eGFR) and lowers *de novo* donor‐specific antibodies (dnDSA) at an acceptable cellular‐rejection risk [9]. Matched‐cohort data link conversion to better graft survival [10], and late conversion, often retaining a low‐dose CNI, has become the preferred way to use the drug [11].

This evidence, like the Cochrane and other systematic reviews, is limited to kidney‐alone recipients [12, 13, 14]. For SPK, the published conversion experience amounts to two case reports, a small early‐rescue series, a late‐conversion cohort, and one matched case‐control study—roughly two dozen patients [15, 16, 17, 18, 19]. For LK it is sparser still: a simultaneous LK conversion has been reported in essentially one patient, the closest analogue being conversion in kidney‐after‐liver (KAL) recipients, and a national survey confirms that belatacept in liver transplantation is used mainly in LK recipients [20, 21, 22].

Here we report a single‐center experience of belatacept conversion in six SPK and six LK recipients, described and analyzed separately because of their different alloimmune risk, and set against a narrative review of the published SPK and LK conversion literature. We focus less on whether belatacept “works” than on how it can be used safely in these off‐label settings: the timing of conversion, the handling of the CNI, and the kidney, alloimmune, metabolic and infectious trade‐offs that follow.

## 2 Materials and Methods

### 2.1 Study Design, Case Selection and Ethics

We retrospectively identified all adult recipients (aged ≥18 years) of an SPK or LK transplant managed at the University Hospital Zurich who were converted more than 6 months after transplantation from a CNI‐based to a belatacept‐based maintenance regimen; all belatacept starts from November 2012 to January 2026 were screened (Figure S1). Eligible patients had a functioning kidney (no dialysis dependency; eGFR ≥15 mL/min/1.73m^2^), a functioning pancreas (no insulin dependency) and/or liver allograft, were Epstein‐Barr virus (EBV) seropositive, and received at least one dose of belatacept. Because EBV seropositivity is a prerequisite for conversion at our center and the cohort was derived from all belatacept starts rather than from all SPK and LK recipients, otherwise suitable EBV‐seronegative candidates would not have been captured. All 12 participants signed the institutional general consent form of the University Hospital Zurich for further use of health‐related data in pseudonymized form and the responsible ethics committee fully approved the study (BASEC 2019‐01274), which complied with the Declaration of Helsinki.

### 2.2 Standard Immunosuppression and Surgical Technique at Our Center

SPK recipients receive induction with rabbit anti‐thymocyte globulin (ATG) and maintenance with tacrolimus, mycophenolate and prednisone, with early steroid withdrawal since 2011 and individualized reintroduction; patient 3 had received basiliximab. All study SPK transplants belong to the enteric‐drainage era of our program [23]: through a median laparotomy, the pancreas is placed head‐up on the right iliac axis with a donor iliac Y‐graft to the common iliac artery and a side‐to‐side duodeno‐jejunostomy; venous drainage was systemic (inferior vena cava or common iliac vein) in five patients and portal (superior mesenteric vein) in one (patient 3). The kidney is placed extraperitoneally on the left iliac axis with an extravesical ureteroneocystostomy (variants in Table 1). LK recipients follow the kidney protocol: basiliximab induction after living donation or donation after brain death with favorable human leucocyte antigen (HLA) matching, ATG otherwise, and tacrolimus, mycophenolate and prednisone individualized according to tolerance and donor‐specific antibody (DSA) status.

**TABLE 1:** Cohort, transplant and conversion characteristics, by organ group.

Patient (TPL type)	Sex / age at conversion (years)	Underlying disease	TPL year (TPL number)	Induction immunosuppression	Conversion after TPL (months)	Primary indication	Contributing factors	Immunosuppression before conversion (last trough, ng/ml)	CNI/mTOR‐I overlap (months) / (target trough, ng/ml)	Immunosuppression post
**SPK recipients**										
**1 (SPK)**	M / 61	DM type I	2012 (1)	ATG	52	CNI‐associated TMA	Limited graft function due to TMA; Tac switched to CyA before conversion; steroids newly started	CyA, MPA (CyA 55)	1 month (CyA dose halved)	Bela, MPA, PDN
**2 (SPK adrenal)**	F / 49	Polyglandular autoimmune syndrome type II / DM type I	2020 (1)	ATG	11	Extrarenal CNI toxicity (polyneuropathy) with histological CNI injury	Molecular AMR phenotype (MMDx) in pre‐conversion biopsy without DSA (no specific anti‐rejection therapy)	Tac, MPA, hydrocortisone (Tac 6.6)	7 months (3‐4 ng/ml)	Bela, MPA, hydrocortisone
**3 (SPK)**	M / 70	DM type I	2007 (1)	Basiliximab	174	CNI nephrotoxicity	Cardiovascular risk profile; adrenal insufficiency (PDN 10 mg); portal venous drainage	Tac, MPA, PDN (Tac 4.7)	1 month (2‐4 ng/ml)	Bela, MPA, PDN
**4 (SPK)**	M / 48	DM type I	2016 (1)	ATG	75	CNI nephrotoxicity	—	Tac, MPA, PDN (Tac 4.9)	6 months (3‐4 ng/ml)	Bela, MPA, PDN
**5 (SPK en bloc)**	F / 56	DM type I	2011 (2)	ATG	159	caAMR (MMDx; no specific anti‐rejection treatment)	Steroids newly started; MPA increased after Tac withdrawal	Tac, MPA (Tac 6.9)	5 months (3‐4 ng/ml)	Bela, MPA, PDN
**6 (SPK)**	M / 29	DM type I	2023 (1)	ATG	19	caAMR (pre‐treated with methylprednisolone, plasmapheresis, IVIG and rituximab)	—	Tac, MPA, PDN (Tac 7.3)	6 months (3‐4 ng/ml)	Bela, MPA, PDN
**LK recipients**										
**7 (LK)**	M / 51	Unknown	2015 (3)	ATG	91	Failing allograft with intolerance of alternative regimens	Everolimus trial stopped for adverse effects; thrombocytopenia possibly CNI‐related; converted at eGFR 18.5 mL/min/1.73m^2^	Tac, MPA, PDN (Tac 3.7)	4 months (3‐4 ng/ml)^*^	Bela, MPA, PDN
**8 (LK)**	F / 71	ADPKD	2019 (1)	Basiliximab	52	Extrarenal CNI intolerance	—	Tac, MPA, PDN (Tac 2.5^**^)	3 months (3‐4 ng/ml)	Bela, MPA, PDN
**9 (LK)**	F / 40	Primary hyperoxaluria type I	2005 (1)	ATG	223	CNI nephrotoxicity	AZA switched to MPA 5 months before conversion	Tac, MPA, PDN (Tac 6.4)	3 months (3‐4 ng/ml)	Bela, MPA, PDN
**10 (LK)**	F / 51	Hereditary amyloidosis	2016 (1)	Basiliximab	86	caAMR (MMDx) under everolimus‐based regimen (no specific anti‐rejection therapy)	—	EVR, MPA, PDN (EVR 5.3)	3 months (EVR 3‐4 ng/ml)^*^	Bela, MPA, PDN
**11 (LK)**	F / 44	Primary hyperoxaluria type I	2016 (1)	Basiliximab	93	CNI nephrotoxicity	Diarrhea; everolimus continued as maintenance (target 3‐4 ng/ml)	Tac, EVR, PDN (Tac 4.0; EVR 4.0)	1 month (EVR 3‐4 ng/ml continued)	Bela, EVR, PDN
**12 (KAL DBD)**	M / 48	COVID‐19 related liver failure (secondary kidney failure)	2024 kidney (1)	ATG	13	Extrarenal CNI intolerance	Anti‐HLA antibodies directed against the liver donor; prevention of CNI nephrotoxicity	Tac, MPA, PDN (Tac 5.4)	6 months (3‐4 ng/ml)	Bela, MPA, PDN

Abbreviations: ADPKD = autosomal‐dominant polycystic kidney disease; AMR = antibody‐mediated rejection; ATG = anti‐thymocyte globulin; AZA = azathioprine; Bela = belatacept; caAMR = chronic‐active antibody‐mediated rejection; CNI = calcineurin inhibitor; COVID‐19 = coronavirus disease 2019; CyA = cyclosporine; DBD = donation after brain death; DM = diabetes mellitus; DSA = donor‐specific antibody; eGFR = estimated glomerular filtration rate; EVR = everolimus; F = female; IVIG = intravenous immunoglobulin; KAL = kidney‐after‐liver transplantation; LK = liver‐kidney transplantation; M = male; MMDx = Molecular Microscope Diagnostic System; MPA = mycophenolate; mTOR‐I = mammalian target of rapamycin inhibitor; PDN = prednisone; SPK = simultaneous pancreas‐kidney transplantation; SPK adrenal = pancreas transplanted en bloc with the donor left adrenal gland (patient 2); SPK en bloc = en‐bloc transplantation of pediatric double kidneys with bladder patch (patient 5); Tac = tacrolimus; TMA = thrombotic microangiopathy; TPL = transplantation.

Overlap = transient reduced‐dose CNI/mTOR‐I co‐administration after the first belatacept dose; last trough = last measured trough before conversion. All SPK transplants had enteric exocrine drainage (duodeno‐jejunostomy); venous drainage was systemic except in patient 3. Prednisone maintenance after conversion was 5 mg/day (10 mg in patient 3; 7.5 tapered to 5 mg in patient 7); hydrocortisone 20‐25 mg/day in patient 2.

* CNI (patient 7) or everolimus (patient 10) was never withdrawn because belatacept was discontinued first (after 4 months for end‐stage renal disease and after 3 months for an allergic reaction, respectively).

** *Single low value; preceding trough 4.4 ng/ml*.

### 2.3 Indications for Conversion and Definitions

Conversion was clinically indicated, not protocolized. We classified the primary indication as CNI nephrotoxicity (progressive kidney dysfunction with histological or clinical evidence of CNI injury), extrarenal CNI toxicity or intolerance (neurological, gastrointestinal or hematological adverse effects), chronic‐active antibody‐mediated rejection of the kidney allograft (caAMR; histological or molecular diagnosis with or without DSA), CNI‐associated thrombotic microangiopathy (TMA), or a failing allograft with intolerance of alternative regimens; contributing factors were recorded separately (Table 1). In caAMR, belatacept was not used as rejection therapy but as the maintenance regimen we considered most appropriate for an injured graft in the era before anti‐CD38 therapies, in line with the stabilization of function and molecular inflammation reported after conversion in caAMR [24]. We preferred complete CNI withdrawal under belatacept to long‐term CNI minimization, which carries alloimmune risk without eliminating nephrotoxicity; minimization had failed in patient 7 (including an everolimus trial), and several others already had low tacrolimus exposure (Table S1).

### 2.4 Conversion Protocol and CNI Handling

Belatacept was dosed at 5 mg/kg every 2 weeks until week 8 and every 4 weeks thereafter (patients 1–3), or as an initial 10 mg/kg dose followed directly by 5 mg/kg every 4 weeks (patients 4–12). The CNI (everolimus in one patient) was always retained transiently at reduced exposure, targeting a tacrolimus or everolimus trough of 3–4 ng/ml (2–4 ng/ml in patient 3; cyclosporine dose halved in patient 1), and tapered to withdrawal according to graft stability, immunological risk and infections (Figure S2). Maintenance after conversion consisted of belatacept, mycophenolate and low‐dose steroids (everolimus instead of mycophenolate in patient 11). Steroids were newly started in two patients (patients 1 and 5; 5 mg prednisone daily); all others were already on maintenance steroids (hydrocortisone in patient 2). Other small changes are described in Table S1.

### 2.5 Monitoring and Prophylaxis

Monitoring after conversion was individualized rather than protocolized (Table S2). Creatinine was measured at every 4‐weekly infusion during at least the first 6 months in most patients (Table S2) and at least every 3 months thereafter. DSA (OneLambda Luminex single‐antigen beads; mean fluorescence intensity (MFI) ≥500 positive, about 1000–2000 clinically relevant on an individual basis) were assessed before or at conversion (last routine screening), at least once during the first post‐conversion year (median two assessments) and annually thereafter as part of routine anti‐HLA screening (Table S3). Cytomegalovirus (CMV), BK polyomavirus and EBV polymerase chain reaction (PCR) testing was symptom‐driven or infrequent (≤4 tests in the first year) in conversions before 2024 and scheduled monthly during the first 3–6 months in most conversions from 2024 onward. Glycated hemoglobin (HbA1c), lipase and transaminases were measured at clinical discretion; lipase was not part of routine monitoring before 2022. No protocol biopsies were performed, and no patient received *Pneumocystis* or CMV prophylaxis at or after conversion.

### 2.6 Objectives, Outcomes and Literature Search

The primary objective was to describe kidney function before and after conversion in each organ group: eGFR at conversion and 12 months, individual pre‐ (−12 to 0 months) and post‐conversion (0 to 12 months) eGFR slopes and, as a sensitivity analysis, eGFR change over yearly intervals from 36 months before to 24 months after conversion (nearest available creatinine values; Supplementary Materials). Secondary outcomes were DSA dynamics and rejection, HbA1c and pancreatic or hepatic enzymes, infections, and graft failure or death. Follow‐up ended on July 1, 2026. For the narrative review we searched PubMed from inception to July 1, 2026 (search string in the Supplementary Materials) for original reports of belatacept conversion in SPK or LK recipients of any design and hand‐searched reference lists. Because the evidence is limited to small, heterogeneous, uncontrolled studies, we summarized it descriptively alongside our cohorts without pooling, protocol registration or risk‐of‐bias scoring. Eight primary SPK/LK reports met the inclusion criteria [15, 16, 17, 18, 19, 20, 21, 22] (Figure S3).

### 2.7 Statistical Analysis

eGFR was calculated with the 2009 Chronic Kidney Disease Epidemiology Collaboration (CKD‐EPI) creatinine equation without a race coefficient (all participants were White). Given the small groups we performed no hypothesis tests and report no p values. SPK and LK recipients were analyzed separately and descriptively as medians with interquartile range (IQR) or range. Individual eGFR slopes were estimated by ordinary least‐squares regression on the quarterly measurements from months −12 to 0 and 0 to 12 (ml/min/year); the within‐patient change in slope was the post‐ minus the pre‐conversion slope. As exploratory measures of precision, not of significance, we calculated the Hodges‐Lehmann estimate of the within‐patient change with an exact confidence interval (CI) at the closest attainable level and fitted random‐intercept linear mixed models of eGFR on time with a change in slope at conversion, using all quarterly measurements of all 12 patients, separately per organ group and pooled. Analyses used Python 3.11 (SciPy, statsmodels) and GraphPad Prism version 11.0.2.

## 3 Results

### 3.1 SPK Recipients

Six SPK recipients (patients 1–6; four men; median age at conversion 53 years, IQR 48–60) transplanted between 2007 and 2023 were converted a median of 64 months (IQR 27–138; range 11–174) after transplantation for CNI nephrotoxicity (*n* = 2), extrarenal CNI toxicity (*n* = 1), caAMR (*n* = 2) or CNI‐associated TMA (*n* = 1) (Table 1). Induction had been ATG in five and basiliximab in one; the CNI overlap lasted a median of 5.5 months (range 1–7). Median eGFR was 45.4 mL/min/1.73m^2^ (IQR 39.4–60.2) at conversion and 55.5 (46.7–63.2) at 12 months, and the median individual slope changed from −3.9 (IQR −6.4 to 0.0) to +5.6 (2.8 to 10.1) ml/min/year, a median within‐patient change of +11.2 (range −0.6 to +25.3; Hodges‐Lehmann +11.2, exact 96.9% CI −0.6 to +25.3). The mixed‐model estimates were −4.6 (95% CI −10.1 to +0.9) for the pre‐conversion slope and +12.6 (95% CI +2.9 to +22.3) ml/min/1.73m^2^ per year for the slope change (Figure 1A,B; individual values in Table S4). Yearly rates showed decline before conversion, a rise in the first post‐conversion year and no further gain in the second year (Table S5); proteinuria did not demonstrate a consistent change (Figure 1C, Tables S6 and S7).

**FIGURE 1:**
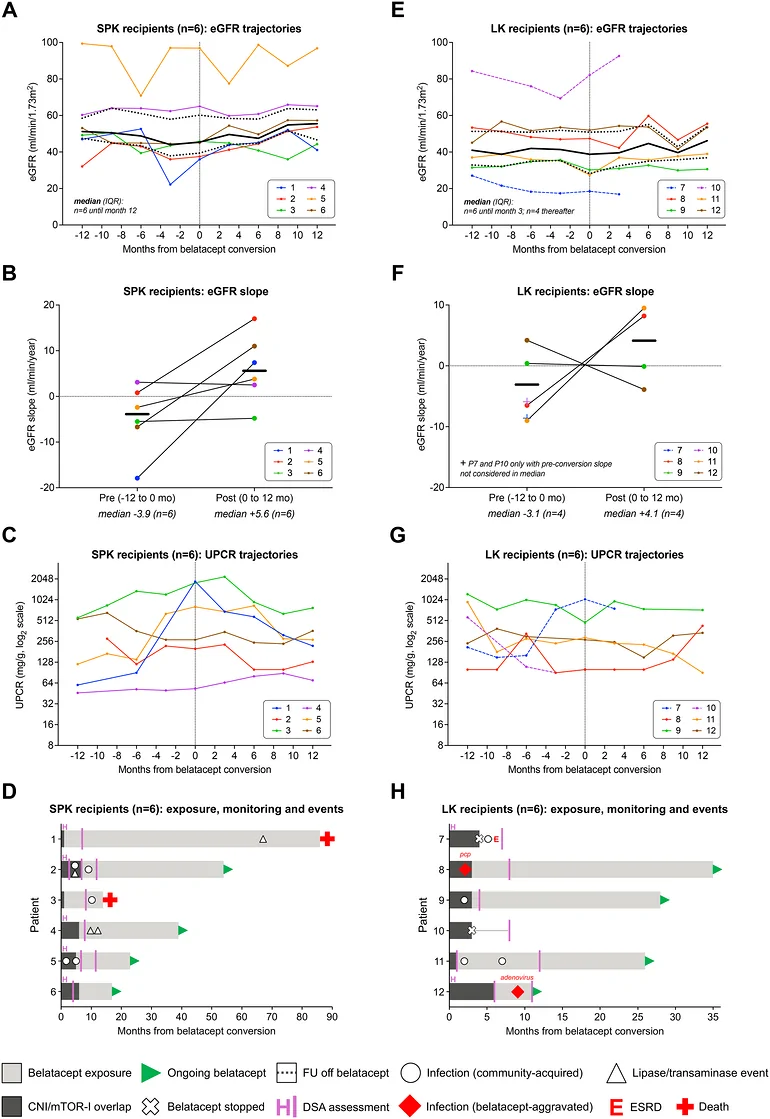
Kidney function, proteinuria and clinical course after belatacept conversion in SPK (A‐D) and LK (E‐H) recipients. (A/E) Individual eGFR trajectories from 12 months before to 12 months after conversion (colored thin lines) with the group median (black thick line) and interquartile range (black dotted lines); dashed lines mark the two LK recipients without a month‐12 value. (B/F) Individual pre‐ (months ‐12 to 0) and post‐conversion (months 0 to 12) eGFR slopes by ordinary least squares, connected per patient, with group medians (horizontal bars); the two LK recipients without a month‐12 value contribute a pre‐conversion slope only. (C/G) Individual urine protein‐to‐creatinine ratio (UPCR) trajectories. (D/H) Timeline per patient from conversion to last follow‐up: bar = belatacept exposure, dark segment = CNI/mTOR‐inhibitor overlap, symbols = DSA assessments, infections, lipase or transaminase events, discontinuation of belatacept, graft loss and death. eGFR by CKD‐EPI 2009. *Abbreviations: CNI = calcineurin inhibitor; DSA = donor‐specific antibody; eGFR = estimated glomerular filtration rate; ESRD = end‐stage renal disease; FU = follow‐up; LK = liver‐kidney transplantation; mTOR‐I = mammalian target of rapamycin inhibitor; PCP = Pneumocystis jirovecii pneumonia; SPK = simultaneous pancreas‐kidney transplantation; UPCR = urine protein‐to‐creatinine ratio*.

DSA at conversion were present in one SPK recipient (anti‐DP6, MFI 1551, patient 4), rose transiently to MFI 4426 and later fell to MFI 615; patient 5 showed a transient, low‐level anti‐DR15 that reappeared and resolved spontaneously. No new DSA developed, and the only post‐conversion biopsy (patient 3) showed no rejection (Tables 2, S3). Pancreatic endocrine function stayed stable: median HbA1c was 5.4% (5.2–5.5) before and 5.3% (5.1–5.5) after conversion, and no recipient became insulin‐dependent. Transient, isolated lipase elevations without pancreatitis occurred in patients 2 (68 U/l) and 4 (62 and 73 U/l; reference 13–60 U/l) within the first year; lipase was not routinely measured in patient 1 during that period. Patient 1 developed a lipase rise to 106 U/l more than 5 years after conversion; because pancreas rejection could not be excluded (no biopsy), it was treated empirically with three pulses of methylprednisolone (250 mg), after which lipase fell and HbA1c stayed ≤5.8%. No viremia and no opportunistic infection occurred; infections were community‐acquired (gastroenteritis, coronavirus disease 2019, influenza, urinary‐tract infections). Two patients died with functioning grafts from cardiovascular causes, 14 (patient 3) and 86 months (patient 1) after conversion; no pancreas or kidney graft was lost, and 4/6 remained on belatacept at a median follow‐up of 31 months (range 14–86; Figure 1D).

**TABLE 2:** Kidney, alloimmune, metabolic, infectious and survival outcomes after conversion, by patient.

Patient (TPL type)	eGFR at conversion / month 12	eGFR slope pre/post (ml/min/year)	DSA at conversion (MFI) and course	New DSA after conversion	Post‐conversion biopsy	HbA1c pre / post (%)	Lipase (SPK) or transaminase (LK) events	Infections (possibly belatacept‐aggravated in bold)	Graft loss / death (months after conversion)	On belatacept at last FU / total FU (months)
**SPK recipients**										
**1 (SPK)**	36.0 / 41.1	−17.9 / +7.4	None	No	None	5.3 / 5.2	Not measured in year 1; lipase 106 U/l at >5 years, 3×250 mg methylprednisolone, then 63 U/l^*^	None	Death (86)	No (death) / 86
**2 (SPK adrenal)**	37.5 / 53.7	+0.8 / +17.0	None	No	None	5.2 / 4.8	Lipase 68 U/l (transient)	Gastroenteritis, COVID‐19	—	Yes / 54
**3 (SPK)**	45.8 / 44.3	−5.5 / ‐4.8	None	No	Yes (no rejection)	5.5 / 5.0	No	Influenza	Death (14)	No (death) / 14
**4 (SPK)**	65.0 / 65.1	+3.1 / +2.5	DP6 1551 to 4426 to 615	No	None	5.5 / 5.7	Lipase 62 and 73 U/l (transient, months 8 and 11); later slight rise during appendicitis^**^	None	—	Yes / 39
**5 (SPK en bloc)**	96.9 / 96.8	−2.4 / +3.8	None (historical DR15; transient DR15 0 to 1245 to 1131 to 0)^***^	No^***^	None	4.9 / 5.4	No	Urinary‐tract infection (x2)	—	Yes / 23
**6 (SPK)**	45.0 / 57.3	−6.7 / +11.0	None	No	None	5.7 / 5.5 (once 6%)	No	None	—	Yes / 17
**LK recipients**										
**7 (LK)**	18.5 / NA	−8.6 / NA	DQ6 2165 to 1319	No	None	5.1 / 4.8	No	Animal‐bite soft‐tissue infection	Graft loss / ESRD (6; after belatacept stop)	No (ESRD) / 4 (6)^****^
**8 (LK)**	47.4 / 55.5	−6.5 / +8.2	None	No	None	5.6 / 5.4	No	**PCP** (during overlap; nosocomial cluster)	—	Yes / 35
**9 (LK)**	30.2 / 30.6	+0.4 / ‐0.1	None	No	None	5.1 / 5.2	No	Upper‐respiratory‐tract infection	—	Yes / 28
**10 (LK)**	82.1 / NA	−5.9 / NA	DQ2 4731 to 2050; DR52 2267 stable	No	Yes (no rejection)^*****^	6.4 / 6.6	No	None	—	No (allergy) / 3
**11 (LK)**	27.5 / 39.0	−9.0 / +9.5	DQ8 2028 to 1391	No	None	4.4 / 4.9	No	Diarrhea without focus; low‐level EBV reactivation	—	Yes / 26
**12 (KAL DBD)**	52.0 / 53.5	+4.2 / ‐3.9	None against kidney donor^******^	No	None	5.2 / 5.6	No	**Adenovirus**	—	Yes / 11

Abbreviations: COVID‐19 = coronavirus disease 2019; DBD = donation after brain death; DSA = donor‐specific antibody; EBV = Epstein‐Barr virus; eGFR = estimated glomerular filtration rate (ml/min/1.73m2, CKD‐EPI 2009); ESRD = end‐stage renal disease; FU = follow‐up; HbA1c = glycated hemoglobin; KAL = kidney‐after‐liver transplantation; LK = liver‐kidney transplantation; MFI = mean fluorescence intensity; NA = not available; PCP = Pneumocystis jirovecii pneumonia; SPK = simultaneous pancreas‐kidney transplantation; TPL = transplantation.

*Slopes by ordinary least squares over months ‐12 to 0 and 0 to 12. Lipase reference range 13‐60 U/l*.

* *Possible pancreas rejection (no biopsy; no rise in HbA1c or glucose)*.

** *Spontaneous resolution after appendectomy*.

*** *Transient, low‐level anti‐DR15 that reappeared and resolved spontaneously, not regarded as a persistent new DSA*.

**** *4 months on belatacept; ESRD (persistent eGFR <15 mL/min/1.73m^2^) 2 months after belatacept stop*.

***** *Biopsy shortly after termination of belatacept because of an allergic reaction*.

****** *Anti‐HLA antibodies directed against the liver donor (different donor); DSA refer to the kidney donor throughout*.

### 3.2 LK Recipients

Six LK recipients (patients 7–12, including one KAL recipient; two men; median age 50 years, IQR 45–51) transplanted between 2005 and 2024 were converted a median of 89 months (IQR 61–93; range 13–223) after the kidney transplant for CNI nephrotoxicity (*n* = 2), extrarenal CNI toxicity or intolerance (*n* = 2), caAMR under an everolimus‐based regimen (*n* = 1) or a failing allograft with intolerance of alternative regimens (*n* = 1) (Table 1); three had new‐onset diabetes after transplantation before conversion. Induction had been ATG in three and basiliximab in three; the overlap lasted a median of 3 months (range 1–6), and in patients 7 and 10 the CNI or everolimus was never withdrawn because belatacept was stopped first. Patient 7, a third kidney transplant converted at an eGFR of 18.5 mL/min/1.73m^2^, reached end‐stage renal disease (ESRD) 6 months after conversion, and patient 10 stopped belatacept after 3 months for an allergic reaction; both lacked a month‐12 eGFR. In the four recipients with 12‐month data, median eGFR was 38.8 mL/min/1.73m^2^ (IQR 29.5–48.6) at conversion and 46.3 (36.9–54.0) at 12 months, and the median slope changed from −3.1 to +4.1 mL/min/year (median within‐patient change +7.1, range −8.1 to +18.5; no exact CI attainable at *n* = 4). The mixed model including all six LK recipients estimated a pre‐conversion slope of −3.1 (95% CI −8.7 to +2.4) and a slope change of +6.3 (95% CI −4.2 to +16.7) ml/min/year (Figure 1E,F); the two recipients without 12‐month data had pre‐conversion slopes of −8.6 and −5.9. Yearly rates showed the same pattern (Table S5), and proteinuria did not change appreciably (Figure 1G).

DSA at conversion were present in three LK recipients and declined during follow‐up: anti‐DQ6 from MFI 2165 to 1319 (patient 7), anti‐DQ2 from MFI 4731 to 2050 with stable anti‐DR52 (patient 10) and anti‐DQ8 from MFI 2028 to 1391 (patient 11). Patient 12 had anti‐HLA antibodies directed against his liver donor but none against the kidney donor. No new DSA developed, and the only post‐conversion biopsy (patient 10, after belatacept had been stopped) showed no rejection (Tables 2, S3). Liver function remained stable without relevant transaminase elevation, and HbA1c was largely stable. Patient 11 had a low‐level EBV reactivation (peak 37 copies/ml) that resolved without intervention; no CMV or BK viremia occurred. Two infections were judged possibly belatacept‐associated or aggravated and were treated successfully: *Pneumocystis jirovecii* pneumonia (PCP) in patient 8 during the CNI overlap, coinciding with a nosocomial PCP cluster at our hospital that also affected patients not receiving belatacept, and an adenovirus infection in patient 12. Other infections (animal‐bite soft‐tissue infection, upper‐respiratory‐tract infection, diarrhea) were self‐limited or treated with antibiotics. One kidney graft was lost (patient 7); no patient died and no liver graft was lost. Belatacept was discontinued in two (ESRD, allergic reaction), and 4/6 remained on belatacept at a median follow‐up of 19 months (range 3–35; Figure 1H).

### 3.3 Pooled Exploratory Analyses

Across the 10 recipients with 12‐month data, the Hodges‐Lehmann estimate of the within‐patient slope change was +8.6 (95% CI +0.1 to +17.4) and of the absolute eGFR change +5.4 (95% CI +0.1 to +10.2) ml/min/1.73m^2^; the pooled mixed model (all 12 patients) estimated a pre‐conversion slope of −3.9 (95% CI −7.8 to −0.1) and a slope change of +9.9 (95% CI +2.9 to +17.0) ml/min/year (Figure S4). Median yearly eGFR rates over the five intervals from 36 months before to 24 months after conversion were −5.6, −2.8, −5.7, +5.6 and +0.8 mL/min/year (Table S5). No malignancy occurred; overall, 8/12 (67%) remained on belatacept at a median follow‐up of 25 months (IQR 13–36; maximum 86).

## 4 Discussion

In this single‐center series we converted six SPK and six LK recipients, including one KAL recipient, from CNI‐ to belatacept‐based immunosuppression, mainly for CNI nephrotoxicity, CNI intolerance and caAMR. We analyzed the two groups separately because the pancreas and the liver place their kidney co‐allograft at opposite ends of the alloimmune risk spectrum. To our knowledge this is the first dedicated series in simultaneous LK recipients. In both groups, introduced late as a conversion rather than a *de novo* regimen and with a transient low‐dose CNI overlap targeting a trough of 3–4 ng/ml, belatacept was feasible, was associated with stable‐to‐improved kidney function and produced no pancreatic or hepatic safety signal.

Our six SPK patients add to roughly two dozen published conversions (Table 3). Mujtaba et al. reported creatinine stabilization without rejection after tacrolimus withdrawal in two pancreas recipients [15]; Esposito et al. added belatacept to low‐dose tacrolimus as early rescue in four SPK recipients without rejection or dnDSA [16]; Masset et al. converted six SPK recipients at a median of 31 months while retaining low‐dose tacrolimus or everolimus, with an eGFR gain of about 20 mL/min and no rejection [17]; and Perrier et al. saw improvement in four SPK recipients but rejection when the CNI was stopped or adherence failed [18]. Chen et al., in the only matched study, started belatacept when the eGFR approached 20 mL/min/1.73m^2^, observed two pancreas rejections and suggested that earlier introduction might improve outcomes [19]. Read together with CTOT‐15 [5], these reports converge on the pattern we followed: late conversion, a retained low‐dose CNI during the transition and a functioning pancreas at baseline. The same group has described monthly basiliximab infusions as rescue maintenance in pancreas recipients intolerant of standard agents [25] and a CNI‐free basiliximab‐belatacept regimen with eculizumab for TMA after SPK transplantation [26]; their place relative to belatacept conversion remains to be defined.

**TABLE 3:** Published reports of belatacept use in SPK and LK recipients (narrative review), with the present cohorts.

Study (year) (reference)	Design	n (SPK / LK)	Indication and timing	CNI handling	Key kidney and rejection outcomes	Follow‐up
Mujtaba 2014 [15]	Case report	1 SPK (+1 PTA)	CNI toxicity; early to late	Tac withdrawn	Creatinine stable; no rejection	>12 months
Esposito 2024 [16]	Case series	4 SPK	Gastroparesis / DGF; early (days 15‐170)	Added to low‐dose Tac	Creatinine improved; no rejection or dnDSA	3‐96 months
Masset 2024 [17]	Cohort series	6 SPK	Kidney/pancreatic dysfunction; late (median 31 months)	Low Tac/EVR + low MPA	eGFR +20 mL/min; no rejection or dnDSA	≥18 months
Perrier 2025 [18]	Case series	5 SPK	CNI toxicity/rejection; median 25 months	Stopped in 2, low‐dose in 3	eGFR 36 to 56 mL/min; rejection at CNI stop / non‐adherence	2‐8 years
Chen 2025 [19]	Matched case‐control	8 SPK (+3 PAK, +10 PTA)	CNI sparing/avoidance; late (low eGFR)	Spared in most; withdrawn in 1	Delta‐eGFR +7% SPK; 2 pancreas rejections; survival as controls	Approximately 10 years
LaMattina 2014 [20]^*^	Case series	0 / 7 liver (HCV+)	Peri‐operative kidney dysfunction; bridge	Belatacept bridge to CNI	Kidney recovery; 1 rejection; 86% 6‐month survival	6+ months
Cristea 2021 [21]	Case series	0 / 8 KAL (sequential)	CNI avoidance at kidney transplantation	Transient low Tac to withdraw	No kidney or liver rejection; no dnDSA	Median 16 months
Dumortier 2026 [22]	National survey	LK‐predominant	Practice survey (France), no exact data on timing	Mostly late switch; CNI minimize/withdraw	Belatacept in LT used mainly after LK; late conversion favored	NA
**Present study, SPK**	**Retrospective cohort**	**6 SPK**	**CNI nephrotoxicity/toxicity, caAMR, TMA; late (median 64 months)**	**Transient low‐dose overlap (target trough 3‐4 ng/ml)**	**eGFR slope ‐3.9 to +5.6 mL/min/year; no new DSA; no rejection detected; pancreatic function stable**	**Median 31 months**
**Present study, LK**	**Retrospective cohort**	**6 LK (incl. 1 KAL)**	**CNI nephrotoxicity/intolerance, caAMR, failing graft; late (median 89 months)**	**Transient low‐dose overlap (target trough 3‐4 ng/ml)**	**eGFR slope ‐3.1 to +4.1 mL/min/year (n = 4); pre‐existing DSA declined; no rejection detected; liver function stable**	**Median 19 months**

Abbreviations: caAMR = chronic‐active antibody‐mediated rejection; CNI = calcineurin inhibitor; DGF = delayed graft function; dnDSA = de novo donor‐specific antibody; DSA = donor‐specific antibody; eGFR = estimated glomerular filtration rate; EVR = everolimus; HCV = hepatitis C virus; KAL = kidney‐after‐liver transplantation; LK = liver‐kidney transplantation; LT = liver transplantation; MPA = mycophenolate; NA = not available; PAK = pancreas‐after‐kidney transplantation; PTA = pancreas transplant alone; SPK = simultaneous pancreas‐kidney transplantation; Tac = tacrolimus; TMA = thrombotic microangiopathy.

* *No liver‐kidney transplant recipients; liver transplant only, but with transient kidney problems*.

The LK experience is sparser and rests on different biology. A simultaneously transplanted liver reduces cellular and antibody‐mediated injury in the co‐transplanted kidney [6], which lowers the immunological stakes of CNI withdrawal but does not protect a kidney transplanted later from a different donor [7], as in our KAL recipient. The published experience consists of belatacept bridging in liver recipients with peri‐operative kidney dysfunction [20], conversion at kidney transplantation in eight KAL recipients without kidney or liver rejection [21], and a French survey showing that belatacept in liver transplantation is used mainly in LK recipients, preferentially as late conversion [22]. Our six patients, with stable liver function and no rejection, are consistent with this small evidence base but cannot fully establish safety. The regulatory warning for the liver derives from a *de novo* trial in which belatacept‐based primary immunosuppression, with or without brief tacrolimus, was itself associated with excess death, graft loss and PTLD and, in the CNI‐free arms, more rejection [8]; this signal is mechanistically distinct from the pancreas signal in CTOT‐15, which tracked CNI cessation. Neither trial tested late conversion of a stable graft with a tapered overlap; their indirect lessons are to avoid *de novo* use and abrupt withdrawal and to convert only once the non‐kidney graft is stable. On this basis belatacept need not be categorically withheld from EBV‐seropositive LK recipients with a clear indication and stable liver function.

The kidney‐function signal must be read as an association. In both groups the eGFR slope reversed after conversion, with confidence intervals excluding no change in SPK but not in LK recipients, and the pooled estimates lie within the range reported for late kidney‐alone conversion [9, 10, 11]. Several features argue against attributing this to belatacept. Patients were converted when the eGFR was falling or after an acute insult, so regression to the mean and resolution of the trigger contribute; the yearly rates show a gain in the first year and stability rather than further improvement in the second, consistent with removal of an injury rather than a sustained drug effect; and the two LK recipients without 12‐month data would have biased the paired analyses toward better outcomes, which is why the mixed models include them. Above all, our design cannot separate the effect of removing the CNI from an effect of belatacept: the drug enables a complete and immunologically safe CNI elimination, and the benefit likely reflects that elimination. External benchmarks rather than controls frame the signal: in caAMR, conversion was associated with a rise in eGFR from 34 to 39 mL/min/1.73m^2^ at 12 months while propensity‐matched CNI‐treated controls declined from 36 to 33 [24], and in the matched SPK study delta‐eGFR improved by 7% [19]. We assembled no contemporaneous control group because conversion was indicated by CNI toxicity, TMA or caAMR; a non‐converted recipient with the same indication would either not exist at our center or differ systematically, and incidence‐density matching cannot remove this confounding by indication.

The alloimmune course was reassuring but short. No new DSA developed and pre‐existing DSA remained stable, in line with kidney‐alone data [9, 10, 11] and with the molecular improvement reported after conversion in caAMR [24]; we regard this as hypothesis‐generating, because single‐antigen‐bead values are noisy, three patients were converted for caAMR and no protocol biopsies were performed. Our use of belatacept in caAMR reflects the view that, before anti‐CD38 therapies, it offered the most appropriate maintenance for an injured graft, not a treatment of rejection; the observed stabilization does not prove that view.

Conversion appeared metabolically safe in SPK recipients: HbA1c stayed stable, no recipient became insulin‐dependent, and only transient, isolated lipase elevations occurred within 12 months, although lipase was not routinely measured before 2022. One SPK recipient developed a clinically suspected, non‐biopsy‐confirmed rejection episode about 6 years after conversion, managed empirically with corticosteroids: the pancreas remains at risk, and our findings apply only to grafts functioning at conversion. The metabolic rationale is nonetheless attractive, because CNIs, especially tacrolimus, are diabetogenic and the pancreatic graft exists to maintain euglycemia [27]. HbA1c and lipase should be monitored in SPK recipients and transaminases in LK recipients, with sustained elevation prompting early evaluation for rejection, pancreatitis, biliary complications, infection or drug toxicity.

The main trade‐off is infection, and both possibly belatacept‐related opportunistic infections occurred in LK recipients. We saw no CMV or BK viremia, but no patient received *Pneumocystis* or CMV prophylaxis, and one LK recipient developed PCP during the overlap. That case coincided with a nosocomial PCP cluster at our hospital, which weakens its attribution to belatacept, yet in a cohort this small it remains a safety signal and argues for considering *Pneumocystis* prophylaxis during the overlap period, when net immunosuppression is highest. Conversion should be accompanied by viral surveillance and individualized minimization of net immunosuppression.

Several limitations persist. This was a small, retrospective, single‐center study of highly selected long‐term survivors with functioning non‐kidney grafts, converted a median of 5–7 years after transplantation but over a wide range (11–223 months; three within 2 years), which limits generalizability and introduces selection and survivor bias. Conversion was clinically indicated rather than protocolized, overlap strategies varied, and concomitant changes (steroid initiation in two, an antimetabolite increase in one) may have contributed to the observed trajectories. Monitoring was individualized and evolved over time, follow‐up was uneven, and the slope analyses rest on few measurements per period, are vulnerable to regression to the mean and, in LK recipients, rest on four patients; the absence of a control group precludes causal inference. All recipients were White, the derivation from belatacept starts may have missed EBV‐seronegative candidates, belatacept use in SPK and LK recipients remains off‐label, and all estimates are exploratory.

In conclusion, late belatacept conversion with a transient low‐dose CNI or everolimus overlap was feasible in selected SPK and LK recipients and was associated with stable‐to‐improved kidney function, no new DSA and no signal of clinically relevant pancreatic or hepatic dysfunction. The strategy appears most appropriate for recipients with a stable non‐kidney allograft, a clear indication and kidney function that is not yet critically low, provided CNI withdrawal is gradual and immunological, metabolic, hepatic, pancreatic and infectious surveillance is maintained. These observations support feasibility rather than efficacy; prospective, multicenter studies are needed to define the optimal timing, degree of CNI minimization and infectious trade‐offs of belatacept conversion in multi‐organ transplantation.

## Author Contributions

Lukas Weidmann and Thomas Schachtner designed and directed the project and performed the analysis. Dusan Harmacek and Lukas Weidmann wrote the manuscript. The other co‐authors assisted with data collection and the review of the manuscript.

## Funding

No specific funding was received for this work.

## Conflicts of Interest

The authors declare no conflicts of interest.

## Use of Artificial Intelligence (AI)

During the preparation of this work the authors used a large language model‐based AI assistant (Anthropic Claude) to support language editing and aid in the selection process of mechanistically fitting statistical analyses. All AI‐assisted outputs were reviewed, verified and edited by the authors, who take full responsibility for the content and conclusions of this work. The AI tool was not used to generate, alter or fabricate any clinical data and is not listed as an author.

## Supporting information




**Supporting File 1**: ctr70719‐sup‐0001‐SupMat.pdf

## Data Availability

The data supporting this study's findings are available on request from the corresponding author, L.W. However, the data are not publicly available due to restrictions, such as the fact that they contain information that could compromise the privacy of research participants.
