# Supplementary material for: Belatacept Conversion in Simultaneous Pancreas‐Kidney and Liver‐Kidney Transplant Recipients: A Single‐Center Feasibility Cohort and Narrative Review
Source: Clin Transplant. 2026 Oct 18;40(10):e70719. doi: 10.1111/ctr.70719 (PMC13677084; doi:10.1111/ctr.70719)

SUPPLEMENTARY MATERIALS

Literature search strategy (PubMed, inception to July 1, 2026). Single sensitive query: (belatacept OR Nulojix OR LEA29Y) AND (pancreas OR «pancreas-kidney» OR «kidney-pancreas» OR SPK OR «simultaneous pancreas» OR liver OR «liver-kidney» OR hepatorenal OR «combined liver» OR multivisceral OR multi-organ OR multiorgan). No article-type filters were applied; reference lists and «cited-by» / «similar-articles» links of relevant reports were screened to snowball additional studies. The evidence was summarized as a narrative review without pooling, protocol registration or risk-of-bias scoring.

Supplementary methods: yearly eGFR change rates. For each patient the nearest available serum creatinine values approximately 36 and 24 months before conversion and 24 months after conversion were retrieved from the electronic record (timing in **Supplementary Table 5**) and converted to eGFR (CKD-EPI 2009, no race coefficient) using age at the sampling date. Yearly change rates were calculated as the eGFR difference divided by the actual time interval in years; the rates for the intervals -12 to 0 and 0 to +12 months are the ordinary least-squares slopes of the quarterly values reported in the main text. The long-term pre-conversion slope is the ordinary least-squares slope over all available values from -36 to 0 months. Values were not available for patients 2, 6 and 12 (transplanted less than 24 months before conversion) and, after conversion, for patients without 24-month follow-up.

Supplementary Table 1. Conversion details, CNI/mTOR-I handling and concomitant immunosuppressive changes, by patient.

Patient (TPL type)	Belatacept dosing scheme	CNI minimization attempted before conversion	Last trough before conversion (ng/ml)	Target trough during overlap (ng/ml)	Overlap (months)	CNI/mTOR-I definitively stopped	Steroids at conversion	Other concomitant changes
**1 (SPK)**	5 mg/kg every 2 weeks to week 8, then every 4 weeks	No (tacrolimus switched to cyclosporine because of TMA)	CyA 55	CyA dose halved after the 2nd belatacept dose	1	19.12.2016	Newly started: PDN 5 mg/d	None
**2 (SPK adrenal)**	5 mg/kg every 2 weeks to week 8, then every 4 weeks	No	Tac 6.6	3-4	7	19.06.2022	Continued: hydrocortisone 25 mg/d (reduced to 20 mg/d retard formulation after conversion)	None
**3 (SPK)**	5 mg/kg every 2 weeks to week 8, then every 4 weeks	No (trough below target 6-8)	Tac 4.7	2-4 (halved after the 2nd infusion)	1	13.01.2022	Continued: PDN 10 mg/d (adrenal insufficiency)	None
**4 (SPK)**	10 mg/kg, then 5 mg/kg every 4 weeks	No	Tac 4.9	3-4	6	01.08.2023	Continued: PDN 5 mg/d	None
**5 (SPK en bloc)**	10 mg/kg, then 5 mg/kg every 4 weeks	No	Tac 6.9	3-4	5	10.10.2024	Newly started: PDN 2.5 mg/d, increased to 5 mg/d	MMF increased from 500 mg to 750 mg twice daily after tacrolimus withdrawal
**6 (SPK)**	10 mg/kg, then 5 mg/kg every 4 weeks	No	Tac 7.3	3-4	6	12.06.2025	Continued: PDN 5 mg/d (since rejection 11/2023)	None
**7 (LK)**	10 mg/kg, then 5 mg/kg every 4 weeks	Yes: tacrolimus target 3-5 ng/ml and switch of EC-MPS to everolimus (target 4-6 ng/ml), stopped for adverse effects (aphthous stomatitis)	Tac 3.7 (target 5-7)	3-4	4 (never withdrawn)	Not stopped (belatacept discontinued after 4 months; ESRD)	Continued: PDN 7.5 mg/d, reduced to 5 mg/d at month 2	None
**8 (LK)**	10 mg/kg, then 5 mg/kg every 4 weeks	No formal minimization (troughs 4-5 at target 5-7)	Tac 2.5 (single low value; 4.4 before)	3-4	3	14.08.2023	Continued: PDN 5 mg/d	None
**9 (LK)**	10 mg/kg, then 5 mg/kg every 4 weeks	No formal minimization (target 4-6 given long-standing transplant)	Tac 6.4	3-4	3	10.03.2024	Continued: PDN 5 mg/d	Azathioprine switched to EC-MPS 5 months before conversion
**10 (LK)**	10 mg/kg, then 5 mg/kg every 4 weeks	Not applicable (everolimus-based regimen)	EVR 5.3	EVR 3-4	3 (never withdrawn)	Not stopped (belatacept discontinued after 3 months; allergic reaction)	Continued: PDN 5 mg/d	None
**11 (LK)**	10 mg/kg, then 5 mg/kg every 4 weeks	Partial (tacrolimus exposure reduced to about 4 ng/ml under tacrolimus plus everolimus)	Tac 4.0; EVR 4.0	EVR 3-4 (CNI tapered faster because of dual CNI/mTOR-I exposure)	1	04.04.2024 (tacrolimus); everolimus continued	Continued: PDN 5 mg/d	Everolimus continued as maintenance (target 3-4 ng/ml) instead of mycophenolate
**12 (KAL DBD)**	10 mg/kg, then 5 mg/kg every 4 weeks	No	Tac 5.4 (target 5-7)	3-4	6	13.01.2026	Continued: PDN 5 mg/d	None

Abbreviations: CNI = calcineurin inhibitor; CyA = cyclosporine; DBD = donation after brain death; EC-MPS = enteric-coated mycophenolate sodium; ESRD = end-stage renal disease; EVR = everolimus; KAL = kidney-after-liver transplantation; LK = liver-kidney transplantation; MMF = mycophenolate mofetil; mTOR-I = mammalian target of rapamycin inhibitor; PDN = prednisone; SPK = simultaneous pancreas-kidney transplantation; Tac = tacrolimus; TMA = thrombotic microangiopathy; TPL = transplantation.

Supplementary Table 2. Monitoring during the first 12 months after conversion, by patient.

Patient (TPL type)	Creatinine / routine laboratory	CMV PCR (schedule; n in year 1)	BK polyomavirus PCR (schedule; n)	EBV PCR (schedule; n)	DSA assessments (months from conversion)	HbA1c (SPK)	Lipase (SPK)	Transaminases (LK)	PCP / CMV prophylaxis
**1 (SPK)**	Every infusion to month 6, then about every 3 months	On suspicion (1: month 0)	On suspicion (2: months 0 and 7)	On suspicion	0, 7	Every 6 months	Not measured (routine lipase only from 2022)	-	None / none
**2 (SPK adrenal)**	Every 4 weeks	Monthly to month 3, then every 6 months (4)	Months 0 and 12 (2)	Months 0, 3 and 12 (3)	0, 3, 7, 12	Months 2, 3, 6, 7, 12	Every 3 months	-	None / none
**3 (SPK)**	Every 4 weeks	On suspicion (1: about month 8)	On suspicion (1)	On suspicion (1)	About 8	Month 4	Month 2	-	None / none
**4 (SPK)**	Every 4 weeks	On suspicion (1: about month 8)	Months 6 and 12 (2)	On suspicion (1: about month 8)	0, 8 (and month 39)	Months 4, 5, 8, 11	Months 0, 8, 10, 11 (later 17, 18)	-	None / none
**5 (SPK en bloc)**	Every infusion to month 6, then about every 3 months	Months 1-6 and 9 (7)	Months 1-6 and 9 (7)	Months 1, 2, 3, 5, 6, 9 (6)	0, 6-7, 11-12	Months 2, 3, 6, 12	Months 2, 3, 6, 8, 12	-	None / none
**6 (SPK)**	Every infusion to month 6, then about every 3 months	Months 0, 1, 2, 4, 5 (5)	Months 0, 1, 2, 4, 5 (5)	Months 0, 1, 2, 4, 5 (5)	0, 4	Months 0, 2, 4	Months 0, 1, 2, 5	-	None / none
**7 (LK)**	Every 4 weeks to month 6, then intensified (ESRD)	Months 0 and 7 (1 on belatacept)	Months 0 and 7 (1 on belatacept)	Months 0 and 7	0, 7	-	-	Months 0, 3, 6	None / none
**8 (LK)**	Every 4 weeks to month 6, then months 8, 10, 12	Months 1 and 3 (2)	Months 1 and 3 (2)	Months 1 and 3 (2)	8	-	-	Months 0, 2, 3, 8, 11	None / none (no secondary prophylaxis after PCP)
**9 (LK)**	Months 1, 2, 4, 6, 8, 10, 12	Months 0, 4, 7 (3)	Months 0, 4, 7 (3)	Months 0, 4, 7 (3)	4	-	-	Months 0, 1, 4, 6, 8, 10, 12	None / none
**10 (LK)**	Every 4 weeks while on belatacept	Months 0 and 3 (2)	Months 0 and 3 (2)	Month 0 (1)	-4 (baseline), 8 (off belatacept)	-	-	Months 0, 3, 7	None / none
**11 (LK)**	Months 0-4, 6, 8, 10, 12	Months 0, 1, 2, 4, 6, 10, 12 (7)	Months 0, 1, 2, 8, 12 (5)	Months 0-4, 7-10, 12 (10; once 37 IU/ml at month 7)	1, 12	-	-	Months 0, 1, 2, 9, 12	None / none
**12 (KAL DBD)**	Months 0, 1, 3, 6, 7, 8, 9, 11	Months 0, 1, 3, 6, 7, 8, 11 (7; once <34.5 IU/ml at month 1)	Months 0, 3, 11 (3)	Months 0, 3, 8, 9, 11 (5)	0, 6, 11	-	-	Months 0, 3, 6, 8, 9, 11	None / none

Abbreviations: BK = BK polyomavirus; CMV = cytomegalovirus; DBD = donation after brain death; DSA = donor-specific antibody; EBV = Epstein-Barr virus; ESRD = end-stage renal disease; HbA1c = glycated hemoglobin; KAL = kidney-after-liver transplantation; LK = liver-kidney transplantation; PCP = Pneumocystis jirovecii pneumonia; PCR = polymerase chain reaction; SPK = simultaneous pancreas-kidney transplantation; TPL = transplantation.

All viral PCR results were negative or below the quantification limit except the low-level EBV reactivation in patient 11. Anti-HLA antibodies are additionally screened annually in all transplant recipients at our center. Monitoring was individualized; scheduled monthly viral PCR during the first months was practiced in conversions from 2024 onward (patients 5, 6, 11, 12).

Supplementary Table 3. Immunological characteristics, DSA dynamics and allograft biopsies.

Patient (TPL type)	HLA mismatches (A/B/DR)	CMV (recipient status)	Pre-conversion kidney biopsy	DSA before conversion (MFI)*	DSA dynamic during/after conversion (MFI)*	New DSA after conversion	Post-conversion biopsy
**1 (SPK)**	4	Positive	Yes (no rejection)	None	No DSA	No	None
**2 (SPK adrenal)**	5	Positive	Yes (molecular AMR phenotype, no DSA)	None	No DSA	No	None
**3 (SPK)**	5	Positive	Yes (no rejection)	None	No DSA	No	Yes (no rejection)
**4 (SPK)**	6	Positive	Yes (no rejection)	DP6 (1551)	DP6 1551 to 4426 to 615	No	None
**5 (SPK en bloc)**	5	Negative	Yes (caAMR)	None (DR15 historical)	DR15 0 to 1245 to 1131 to 0	No**	None
**6 (SPK)**	3	Positive	Yes (no rejection; after primary AMR treatment)	None	No DSA	No	None
**7 (LK)**	3	Positive	None	DQ6 (2165)	DQ6 2165 to 1319	No	None
**8 (LK)**	2	Negative	None	None	No DSA	No	None
**9 (LK)**	5	Positive	Yes (no rejection)	None	No DSA	No	None
**10 (LK)**	6	Positive	Yes (caAMR)	DQ2 (4731); DR52 (2267)	DQ2 4731 to 2050; DR52 stable	No	Yes (no rejection)***
**11 (LK)**	5	Positive	Yes (no rejection)	DQ8 (2028)	DQ8 2028 to 1391	No	None
**12 (KAL DBD)**	4	Positive	None	None against the kidney donor****	No DSA	No	None

Abbreviations: AMR = antibody-mediated rejection; caAMR = chronic-active antibody-mediated rejection; CMV = cytomegalovirus; DBD = donation after brain death; DSA = donor-specific antibody; HLA = human leukocyte antigen; KAL = kidney-after-liver transplantation; LK = liver-kidney transplantation; MFI = mean fluorescence intensity; SPK = simultaneous pancreas-kidney transplantation; TPL = transplantation.

**Last Luminex single-antigen class I and II test before conversion considered. Specific re-typing for HLA loci other than A/B/DR (e.g., C, DQ or DP) was performed if relevant anti-HLA antibodies occurred in the Luminex test.*

***Patient 5 showed a transient, low-level anti-DR15 that re-appeared and resolved spontaneously to zero, not regarded as a persistent new DSA.*

****Biopsy shortly after termination of belatacept because of an allergic reaction.*

*****Anti-HLA antibodies directed against the liver donor (different donor) were present; DSA in this table refer to the kidney donor.*

Supplementary Table 4. eGFR values (ml/min/1.73m^2, CKD-EPI 2009) for all individuals at all time points (-12 months to +12 months).

Patient	-M12	-M9	-M6	-M3	Bela start	M3	M6	M9	M12
**1 (SPK)**	47	-	52.6	22.2	36	43.8	45.2	52.1	41.1
**2 (SPK adrenal)**	32.1	44.8	43.1	36	37.5	41.2	44.5	51.4	53.7
**3 (SPK)**	49.2	50.5	39.4	43.5	45.8	44.9	40.8	36	44.3
**4 (SPK)**	60.3	64	63.9	62.4	65	59.8	60.8	65.9	65.1
**5 (SPK en bloc)**	99.4	97.9	70.9	97	96.9	77.5	98.7	87.2	96.8
**6 (SPK)**	53.1	45	44.9	44.5	45	54.4	49.7	57.4	57.3
**7 (LK)**	27.1	21.6	18.3	17.4	18.5	16.9	-	-	-
**8 (LK)**	53.4	51.3	48.2	47	47.4	42.3	59.8	46.7	55.5
**9 (LK)**	31.5	32.1	34.7	35.7	30.2	31	32.7	30	30.6
**10 (LK)**	84.3	-	76	69.4	82.1	92.6	-	-	-
**11 (LK)**	37	38.8	35.9	35.3	27.5	36.9	35.7	37.7	39
**12 (KAL DBD)**	45.1	56.7	51.7	53.5	52	54.3	53.7	41.6	53.5

Supplementary Table 5. eGFR change per year over five yearly intervals from 36 months before to 24 months after conversion, by patient.

Patient	eGFR 36 months before conversion (actual timing, months)	eGFR 24 months before conversion (actual timing, months)	eGFR 24 months after conversion (actual timing, months)	-36 to -24 months	-24 to -12 months	-12 to 0 months (pre-conversion slope)	0 to +12 months (post-conversion slope)	+12 to +24 months	Long-term pre-conversion slope (-36 to 0 months)
**1 (SPK)**	49.2 (-35)	60.8 (-29)	73.6 (+25)	+22.7	-9.9	-17.9	+7.4	+30.8	-7.3
**2 (SPK adrenal)**	-	-	49.9 (+23)	-	-	+0.8	+17.0	-4.2	-
**3 (SPK)**	62.1 (-37)	65.4 (-25)	-	+3.5	-14.6	-5.5	-4.8	-	-7.7
**4 (SPK)**	69.3 (-37)	63.2 (-25)	59.6 (+24)	-6.0	-2.8	+3.1	+2.5	-5.5	-1.4
**5 (SPK en bloc)**	101.3 (-39)	100.1 (-28)	93.1 (+23)	-1.3	-0.5	-2.4	+3.8	-4.1	-3.4
**6 (SPK)**	-	-	-	-	-	-6.7	+11.0	-	-
**7 (LK)**	31.9 (-34)	27.3 (-25)	-	-5.9	-0.2	-8.6	-	-	-5.1
**8 (LK)**	54.2 (-37)	48.7 (-25)	56.7 (+23)	-5.6	+4.3	-6.5	+8.2	+1.3	-1.7
**9 (LK)**	34.5 (-36)	27.9 (-24)	31.2 (+22)	-6.2	+3.7	+0.4	-0.1	+0.8	+0.2
**10 (LK)**	77.3 (-34)	99.4 (-24)	-	+25.1	-15.5	-5.9	-	-	-3.4
**11 (LK)**	69.4 (-38)	51.6 (-24)	43.5 (+27)	-15.8	-14.6	-9.0	+9.5	+3.7	-12.4
**12 (KAL DBD)**	-	-	-	-	-	+4.2	-3.9	-	-
**Median SPK**				+1.1 (n=4)	-6.4 (n=4)	-3.9 (n=6)	+5.6 (n=6)	-4.1 (n=4)	-5.4 (n=4)
**Median LK**				-5.9 (n=5)	-0.2 (n=5)	-6.2 (n=6)	+4.1 (n=4)	+1.3 (n=3)	-3.4 (n=5)
**Median all**				-5.6 (n=9)	-2.8 (n=9)	-5.7 (n=12)	+5.6 (n=10)	+0.8 (n=7)	-3.4 (n=9)

Rates in ml/min/year; eGFR in ml/min/1.73m^2 (CKD-EPI 2009). Historical and 24-month values are the nearest available serum creatinine values (actual timing relative to conversion in parentheses); not available for patients 2, 6 and 12, who were transplanted less than 24 months before conversion, and after conversion for patients without 24-month follow-up. The -12 to 0 and 0 to +12 month rates are the ordinary least-squares slopes of the quarterly values (main text).

Supplementary Table 6. Proteinuria as urine protein-to-creatinine ratio (UPCR, mg/g creatinine) for all individuals at all time points (-12 months to +12 months).

Patient	-M12	-M9	-M6	-M3	Bela start	M3	M6	M9	M12
**1 (SPK)**	60	-	90	-	1880	690	580	315	221
**2 (SPK adrenal)**	-	280	120	220	200	230	100	100	130
**3 (SPK)**	562	847	1360	1220	1800	2200	949	640	779
**4 (SPK)**	46	-	52	50	53	65	80	88	70
**5 (SPK en bloc)**	120	170	140	640	810	690	840	280	270
**6 (SPK)**	540	660	360	270	270	350	246	236	363
**7 (LK)**	210	150	160	740	1040	760	-	-	-
**8 (LK)**	100	100	330	90	100	100	100	140	430
**9 (LK)**	1230	740	1020	861	480	970	750	-	727
**10 (LK)**	570	-	110	90	100	-	-	-	-
**11 (LK)**	950	180	280	240	290	240	230	170	90
**12 (KAL DBD)**	240	390	300	-	270	250	150	310	340

Supplementary Table 7. Albuminuria as urine albumin-to-creatinine ratio (UACR, mg/g creatinine) for all individuals at all time points (-12 months to +12 months).

Patient	Bela start	M3	M6	M9	M12
**1 (SPK)**	-	-	470	183	102
**2 (SPK adrenal)**	84	137	13	9	-
**3 (SPK)**	-	1760	-	459	524
**4 (SPK)**	-	5	13	-	5
**5 (SPK en bloc)**	-	616	-	-	149
**6 (SPK)**	188	-	117	117	187
**7 (LK)**	872	651	-	-	-
**8 (LK)**	-	-	-	21	-
**9 (LK)**	-	-	582	242	435
**10 (LK)**	-	-	-	-	-
**11 (LK)**	-	-	-	-	24
**12 (KAL DBD)**	-	-	-	120	163

Supplementary Figure 1. Derivation of the cohort. All belatacept starts from November 2012 until January 2026 were assessed. *Abbreviations: LK = liver-kidney transplantation; SIK = simultaneous islet-kidney transplantation; SPK = simultaneous pancreas-kidney transplantation; TPL = transplantation.*

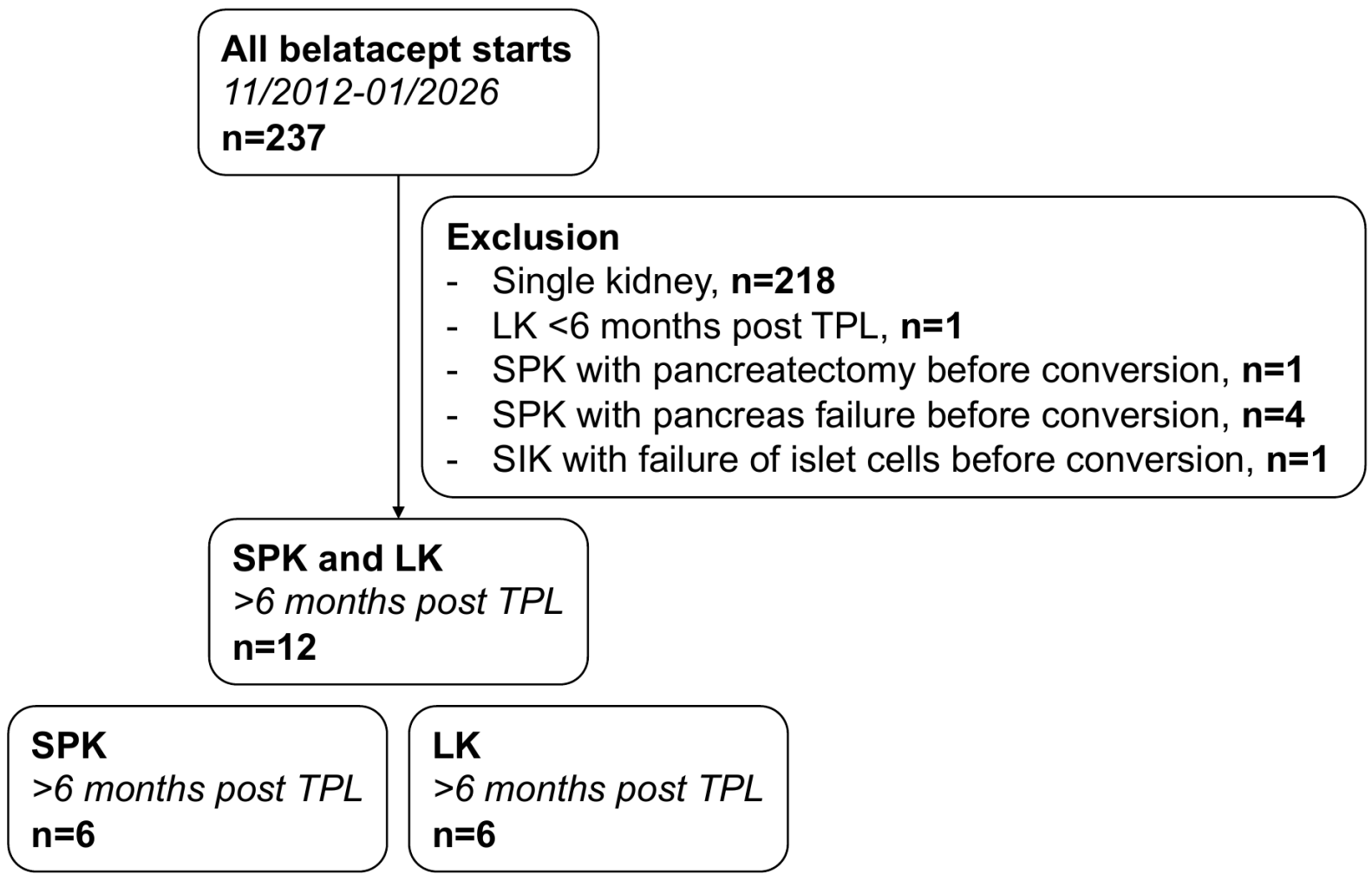

Supplementary Figure 2. Individual eGFR course from belatacept conversion to month 12 with the duration of the transient CNI/mTOR-I overlap. Shaded area = CNI/mTOR-I overlap period (patient 7: CNI never withdrawn, belatacept stopped at month 4, ESRD at month 6; patient 10: everolimus never withdrawn, belatacept stopped at month 3 because of an allergic reaction; patient 11: CNI stopped at month 1, everolimus continued as maintenance). *Abbreviations: CNI = calcineurin inhibitor; DBD = donation after brain death; eGFR = estimated glomerular filtration rate; ESRD = end-stage renal disease; KAL = kidney-after-liver transplantation; LK = liver-kidney transplantation; mTOR-I = mammalian target of rapamycin inhibitor; SPK = simultaneous pancreas-kidney transplantation.*

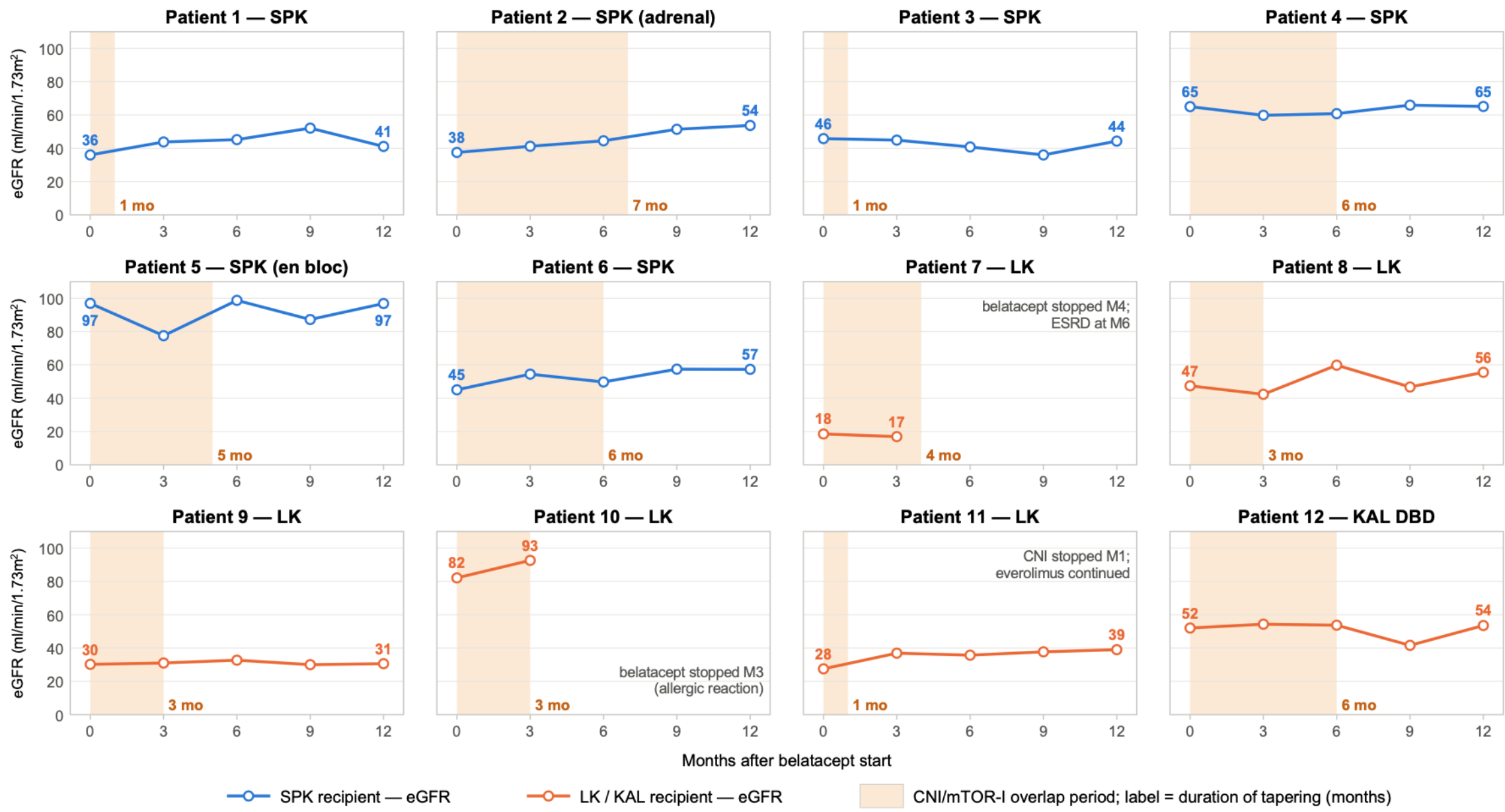

Supplementary Figure 3. PRISMA-style flow diagram of the literature search and study selection for Table 3 (narrative review).

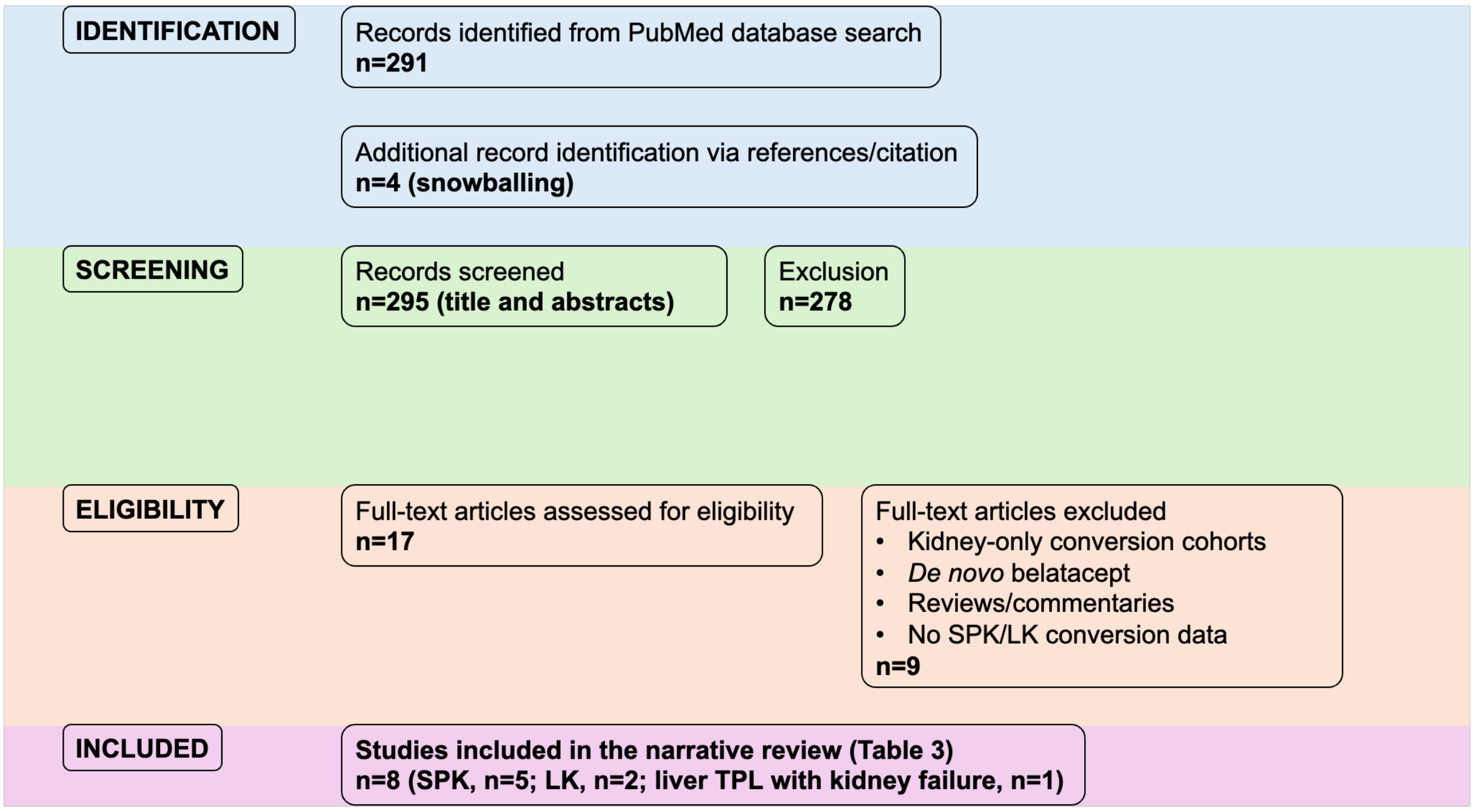

Supplementary Figure 4. Pooled exploratory analyses (all 12 patients). (A) Individual eGFR trajectories (SPK blue, LK orange; dashed = no month-12 value) with the pooled median (thick line) and interquartile range (band). (B) Individual pre- and post-conversion eGFR slopes in the 10 patients with 12-month data, with medians; the Hodges-Lehmann estimate of the within-patient change and the pooled mixed-model estimate are given as exploratory measures of precision. (C) Yearly eGFR change rates over five intervals from 36 months before to 24 months after conversion (points = patients, bars = medians; n per interval indicated; **Supplementary Table 5**). *Abbreviations: CI = confidence interval; eGFR = estimated glomerular filtration rate; HL = Hodges-Lehmann estimate; IQR = interquartile range; LK = liver-kidney transplantation; SPK = simultaneous pancreas-kidney transplantation.*

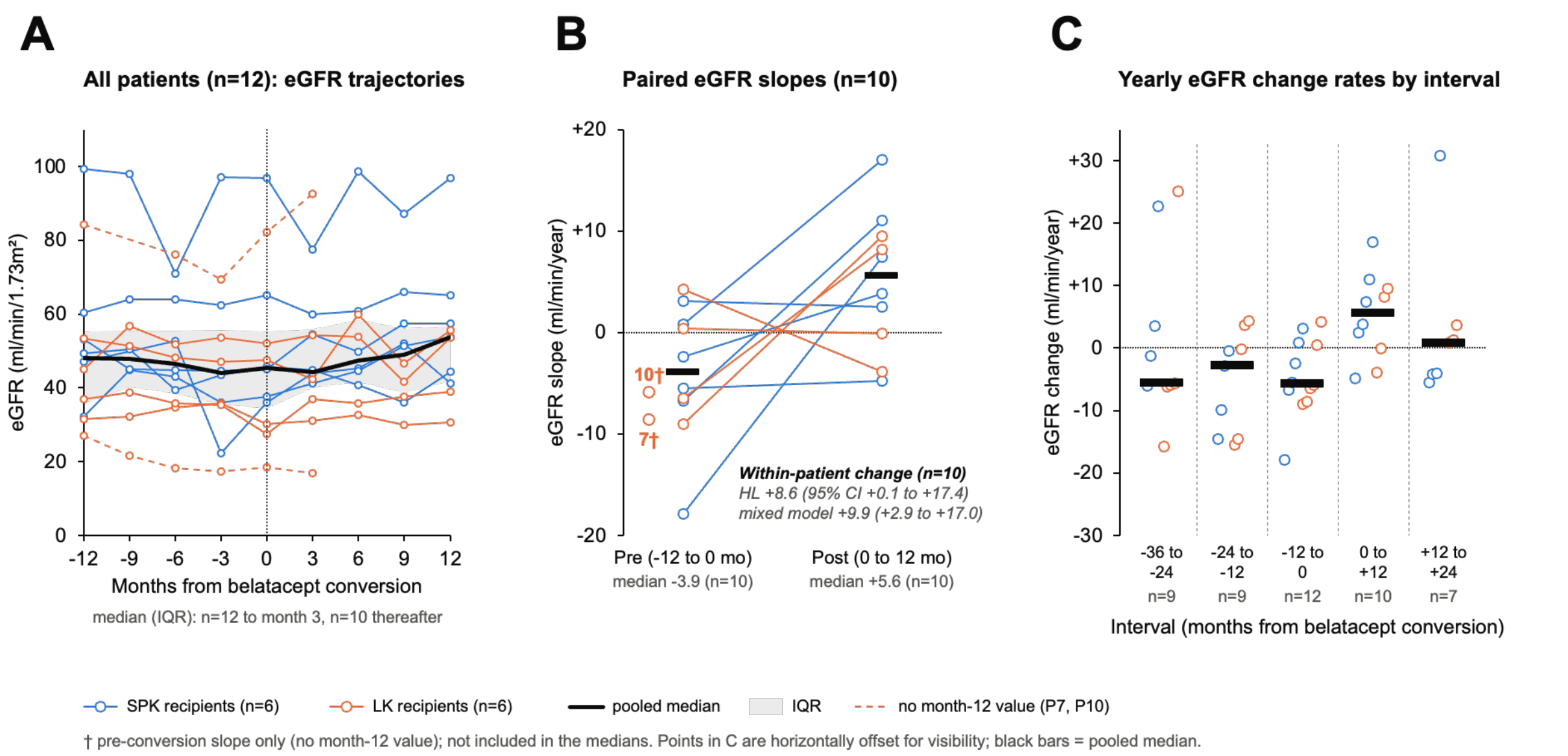

Supplement: Supplementary file 1 [file CTR-40-e70719-s001.pdf]
